# Interactive effects of early *in ovo* feeding and incubation temperature on egg quality traits and embryonic development dynamics in broiler breeders

**DOI:** 10.1016/j.psj.2026.107285

**Published:** 2026-06-12

**Authors:** Maxwell Ansong Okai, Francis Kruenti, Yendouhamtchié Nadiedjoa, Achiamaa Asafu-Adaye Koranteng, Vida Korkor Lamptey, Benjamin. Adjei-Mensah, Jacob Alhassan Hamidu, Kokou Tona, Felix Kwame Amevor, Hai Lin

**Affiliations:** a Key Laboratory of Efficient Utilization of Non-grain Feed Resources (Co-construction by Ministry and Province), Ministry of Agriculture and Rural Affairs, Shandong Provincial Key Laboratory of Animal Nutrition and Efficient Feeding, Department of Animal Science, Shandong Agricultural University, Tai’an, Shandong 271017, China; b Council for Scientific and Industrial Research, Animal Research Institute, P. O. Box AH 20, Achimota, Accra, Ghana; c Department of Animal Science, University of Ghana, P.O. Box LG 25, Legon, Ghana; d Department of Animal Science, Faculty of Agriculture, Kwame Nkrumah University of Science and Technology, Kumasi, Ghana; e Regional Center of Excellence on Avian Sciences at the University of Lomé, Lomé, Togo

**Keywords:** Broiler chicken, Egg quality, Embryonic development, Incubation temperature, *In ovo* feeding

## Abstract

Embryonic growth and nutrient utilization during incubation are influenced by both thermal conditions and nutrient availability; however, information on the combined effects of *in ovo* nutrient supplementation and incubation temperature on embryonic development remains limited. This study investigated the interactive effects of early *in ovo* feeding and incubation temperature on egg quality traits and embryonic development in broiler breeder eggs. A total of 270 fertile eggs were randomly assigned to a 3 × 3 factorial arrangement comprising three *in ovo* treatments (phosphate-buffered saline (PBS; control), glucose, and vitamin D_3_) and three constant incubation temperatures (36.5°C, 37.0°C, and 37.5°C), with three replicates of 10 eggs per treatment combination. On embryonic day (ED) 10, the eggs were injected with 0.2 mL PBS, glucose solution (5 mg/mL), or vitamin D_3_ solution (25 mg/mL). The egg and embryo measurements were recorded daily from ED15 to ED20. Significant *in ovo* treatment × incubation temperature interactions (*p* < 0.05) were observed for several developmental traits. The egg weight was affected only on ED18, while eggshell thickness was influenced on ED15 and ED17-20. In addition, wet embryo weight showed interaction effects on ED15 and ED17-20, whereas femur length was affected on ED15 and ED17, and tibia length on ED16, ED17, and ED19. The results showed that eggshell temperature was influenced on most incubation days except ED16. Wet residual yolk sac weight exhibited an interaction effect only on ED19, while dry residual yolk sac weight was affected from ED17 to ED20 (*p* < 0.05). Incubation temperature significantly influenced embryonic development, with embryos incubated at 37.0°C generally exhibiting lower dry embryo weight and shorter embryo length than those incubated at 36.5°C and 37.5°C. Glucose supplementation increased dry embryo weight on selected days, whereas vitamin D_3_ supplementation enhanced embryonic growth, particularly at 36.5°C. Taken together, embryonic responses to nutrient supplementation were temperature-dependent, with vitamin D_3_-injected eggs incubated at 36.5°C showing the greatest growth and skeletal development, while embryos incubated at 37.0°C exhibited the poorest growth performance.

## Introduction

Genetic selection and advancements in management have markedly accelerated growth rates in modern broiler chickens, substantially shortening the time required to reach market weight (Speier et al., 2012; van der Wagt et al., 2020). As a consequence, embryonic development and hatch quality have become increasingly critical determinants of posthatch performance, survivability, and lifetime productivity. Because avian embryos depend entirely on the finite nutrient reserves deposited in the egg at oviposition, the quantity, composition, and temporal availability of nutrients during incubation directly influence organogenesis, skeletal development, metabolic programming, and early post-hatch viability (Yadgary and Uni, 2012; Dong et al., 2013). Therefore, strategies aimed at optimizing the embryonic environment represent a promising approach to enhance hatch outcomes and subsequent performance.

*In ovo* technology has emerged as an effective prenatal intervention to modulate embryonic nutrition, metabolism, and immune competence. The delivery of carbohydrates, amino acids, vitamins, and minerals into the amniotic or air sac compartments has been shown to influence embryonic growth trajectories, improve hatchability, and enhance early posthatch growth and intestinal development (Uni and Ferket, 2004; Zhai et al., 2011; Bello et al., 2013). Nutrient and mineral supplementation during incubation can improve bone mineralization and skeletal integrity at hatch (Yair et al., 2013; Imran et al., 2023), while antioxidant and metabolic modulators may mitigate oxidative stress associated with incubation challenges (Abuoghaba, 2017). In addition, *in ovo* vaccination is widely implemented in commercial systems, demonstrating that embryonic intervention can be performed safely without compromising hatchability when properly administered (Moura et al., 2007; Peebles et al., 2016, 2017; Peebles, 2018; Sokale et al., 2018).

Among environmental factors, incubation temperature is one of the most critical determinants of embryonic development. Small deviations from the optimal thermal range can alter embryonic metabolic rate, oxygen consumption, tissue accretion, and endocrine responses (Leksrisompong et al., 2007; Maatjens et al., 2014,^2016^; Masia et al., 2025). Temperature profiles during mid to late incubation influence hatching synchrony, chick quality, organ maturation, and physiological competence at hatch (Decuypere and Michels, 1992; Sgavioli et al., 2016). Moreover, incubation temperature affects nutrient utilization efficiency, residual yolk absorption, and skeletal growth, with potential long-term effects on growth performance and early mortality (French, 1997; Agyekum et al., 2022; Tona et al., 2022). The variability in breeder age and egg characteristics further modulates eggshell conductance and heat exchange, thereby influencing embryonic thermal balance and developmental outcomes (Gualhanone et al., 2012).

Despite extensive investigation of *in ovo* nutrition and incubation temperature as independent factors, limited research has evaluated their interactive effects on embryonic development. Because incubation temperature directly modulates embryonic metabolic rate and nutrient turnover, it may alter the efficacy of *in ovo*-administered substrates such as glucose and vitamin D_3_. Glucose serves as a rapidly available energy source during periods of high metabolic demand, particularly in late incubation, whereas vitamin D_3_ plays a pivotal role in calcium metabolism and skeletal mineralization. However, whether their benefits are enhanced or diminished under different thermal conditions remains insufficiently understood (Retes and Oliveira, 2018; Dayan et al., 2020; Yair et al., 2013; Abbasi et al., 2017).

Therefore, the objective of this study was to evaluate the interactive effects of *in ovo* supplementation with glucose or vitamin D₃ and constant incubation temperatures (36.5°C, 37.0°C, and 37.5°C) applied throughout the incubation period on egg quality traits and embryonic development in broiler breeder eggs. We hypothesized that incubation temperature would modulate the physiological response to *in ovo* nutrient administration, resulting in stage-specific differences in embryonic growth, skeletal development, and yolk utilization.

## Materials and methods

### Ethical approval

All experimental procedures were approved by the Animal Care and Use Committee of Shandong Agricultural University (Taian, China) and conducted in accordance with the Guidelines for Experimental Animals issued by the Ministry of Science and Technology of the People’s Republic of China.

### Experimental site and egg source

The experiment was conducted at the Hatchery Unit of the College of Animal Science and Technology, Shandong Agricultural University (Taian, China). Fertilized broiler breeder eggs were obtained from Weifang Hengrui Livestock Co., Ltd. (Weifang, China). Eggs were collected within 24 h of oviposition and transported under standard handling conditions to the hatchery facility. The eggs were stored under commercial hatchery conditions before setting. Upon arrival at the hatchery, eggs were stored at 16-18°C and 70-75% relative humidity for two (2) days before incubation.

### Experimental design

The study was arranged as a 3 × 3 factorial experiment in a completely randomized design with two fixed factors: *In ovo* feeding (IOF): Control (sham injection with sterile phosphate-buffered saline, PBS); Glucose; and Vitamin D_3._ The incubation temperature treatments consisted of constant temperatures of 36.5°C, 37.0°C, and 37.5°C maintained throughout incubation. Three single-stage automatic incubators (Senjie Series DZ47-53) were operated simultaneously, with one incubator assigned to each target temperature. The relative humidity was maintained at 60%, and eggs were automatically turned hourly. A total of 270 fertile eggs were used in the factorial experiment (9 treatment combinations × 30 eggs per combination). Each treatment combination consisted of three replicate groups of 10 eggs. The experimental unit was the individual egg per embryo.

### Egg handling and pre-incubation measurements

Eggs were individually weighed within 24 h post-lay using a precision electronic balance (Ohaus SP602AM; readability 0.1 g). Then the eggs were then randomly allocated to treatments and placed in their designated incubators. The initial egg weight did not differ among treatment groups before incubation (*p* > 0.05), confirming successful randomization.

### *In ovo* injection procedure

The *In ovo* treatments were administered on embryonic day (ED) 10. For each incubation temperature, eggs were randomly assigned to one of the three IOF treatments (3 replicates × 10 eggs per treatment per temperature). Control group: received 0.2 mL sterile PBS (sham injection); Glucose group: received 0.2 mL glucose solution (5 mg/mL); and Vitamin D₃ group: received 0.2 mL vitamin D₃ solution (25 mg/mL). The glucose and vitamin D_3_ dosages were selected based on previous studies reporting positive effects on embryonic development and hatchability (Zhai et al., 2011; Bello et al., 2013; Abbasi et al., 2017). The glucose (analytical grade) was dissolved in sterile PBS. Vitamin D_3_ was solubilized in ethanol and subsequently diluted with PBS to the final concentration. All solutions were prepared under sterile conditions. Injection was performed through the air sac using sterile syringes, following the method described by Zhai et al. (2011). After injection, the shell opening was sealed with sterile wax, and eggs were immediately returned to their respective incubators.

### Sampling schedule

The embryonic and egg component measurements were conducted on ED15, ED16, ED17, ED18, ED19, and ED20. Five (5) eggs per treatment combination were sampled daily, distributed proportionally across replicates. Over six sampling days, all 270 eggs were evaluated (45 eggs/day × 6 days = 270 eggs). On each sampling day, at least one egg was selected from each replicate, and the remaining samples were randomly selected to ensure proportional representation of all replicates.

### *Egg quality measurements (*Egg weight and eggshell temperature)

Eggs were weighed daily using the same electronic balance. Eggshell temperature (EST) was measured at the equator using a digital thermometer at 6-h intervals (morning, afternoon, evening, and night), and mean daily EST was calculated. The eggshell temperature measurements were obtained from all viable eggs present in each treatment combination before daily destructive sampling. The eggshell temperature was measured using a vicks infrared thermometer (model V971 CFN-CAN; temperature range is 32°C to 42.9°C, accuracy within ± 17.7°C) at 06:00am, 12:00pm, 06:00pm, and 12:00am h, and daily means were calculated.

### Eggshell thickness

After embryo removal, shells were air-dried for 24 h at room temperature. Shell thickness (including membranes) was measured at the equator using a micrometer screw gauge (resolution 0.001 mm). Measurements were recorded in triplicate per shell.

### Residual yolk sac (RYS)

The yolk sac was carefully excised, blotted dry, and weighed to determine wet RYS weight. Samples were dried at 65 °C for 4 days in a forced-air oven and reweighed to obtain dry RYS weight.

### Embryo measurements (Embryo temperature, weight, length and bone dimensions)

In this study, the rectal temperature of the embryo was measured between 12:30 and 13:00 h using a veterinary digital thermometer. Furthermore, the embryos were separated from the yolk sac and weighed to obtain the wet embryo weight. Then for the dry weight determination, the embryos were dried at 65 °C for 4 days and reweighed. Embryo length was measured from the tip of the beak to the tip of the middle toe using a divider and ruler, according to Browne (2006).

The femur and tibia bones were removed and immersed in boiling water for 15 min to facilitate soft tissue removal. Bone lengths were measured using a digital vernier caliper.

### Statistical analysis

Data obtained in this study were analyzed using the General Linear Model (GLM) procedure of Minitab (version 19). The statistical model was:

Y_ijk_ = μ + IOF_i_ + TEMP_j_ + (IOF × TEMP)_ij_ + e_ijk_

where Y_ijk_ is the measured trait, µ the overall mean, IOF_i_ the fixed effect of *in ovo* feeding (_i_ = control, glucose, vitamin D3), TEMP_j_ the fixed effect of incubation temperature (_j_ = 36.5, 37.0, 37.5°C), (IOF × TEMP) _ij_ their interaction, and _eijk_ the random residual error. Post-hoc mean comparisons were performed with Tukey’s test and significance declared at α = 0.05. The sample size varied according to the parameter measured. Destructive measurements were based on five (5) eggs per treatment combination per sampling day, whereas repeated measurements such as eggshell temperature involved all available eggs prior to sampling.

## Results

### Egg weight and eggshell thickness

#### Main effects of *in ovo* feeding and incubation temperature

Egg weight showed no significant differences (*p* > 0.05) among *in ovo* treatments (vitamin D_3_, glucose, and control) across most incubation stages (ED15-ED20), except on ED16, where vitamin D₃-injected eggs (64.89 g) were significantly heavier (*p* < 0.05) than glucose-injected (62.85 g) and control eggs (59.03 g). Across all treatments, egg weight declined progressively with incubation age, reflecting normal moisture loss during embryogenesis. Although differences were generally minimal, control eggs tended to retain slightly higher weights than injected groups on most sampling days (Table 1, Table 2, Table 3).

**Table 1:** Effects of incubation temperature and *in ovo* feeding on embryonic development days 15 and 16.

Parameters	Feeding Group	Egg Weight (g)	Eggshell Thickness (mm)	Wet RYS (g)	Dry RYS (g)	Wet Embryo Weight (g)	Dry Embryo Weight (g)	Embryo Length (mm)	Embryo Temperature (°C)	Femur Length (mm)	Tibia Length (mm)
	INCUBATION DAY 15										
*In Ovo* Feeding (IOF)	CON	64.95	0.33	12.22	3.82	13.27	1.25	8.95	35.19^a^	1.06	0.87^a^
	GLU	62.75	0.32	11.14	3.43	12.51	1.01	8.67	34.07^b^	1.04	0.64^b^
	VIT	63.57	0.32	11.31	3.08	11.46	1.23	8.83	33.93^c^	0.93	0.82^ab^
	**SEM**	**0.91**	**0.01**	**0.98**	**0.44**	**0.53**	**0.09**	**0.17**	**0.24**	**0.06**	**0.05**
	***p*-value**	**0.24**	**0.38**	**0.71**	**0.49**	**0.07**	**0.49**	**0.50**	**0.01**	**0.31**	**0.01**
Temperature (Temp) (°C °C)	36.5	64.90	0.33	13.71^a^	3.86	14.00^a^	1.37	9.14^a^	34.87^a^	1.25^a^	0.93^a^
	37.0	63.53	0.32	8.55^b^	3.03	13.25^a^	0.79	8.93^ab^	34.75^a^	0.99^b^	0.65^b^
	37.5	62.85	0.32	12.41^a^	3.45	10.01^b^	1.33	8.39^b^	33.57^b^	0.81^b^	0.75^ab^
	**SEM**	**0.91**	**0.01**	**0.98**	**0.44**	**0.53**	**0.09**	**0.17**	**0.24**	**0.06**	**0.05**
	***p*-value**	**0.29**	**0.99**	**0.02**	**0.41**	**<0.01**	**0.41**	**0.01**	**0.01**	**<0.01**	**0.03**
	**INCUBATION DAY 16**										
*In Ovo* Feeding (IOF)	CON	59.03^c^	0.32	12.03^ab^	3.71	15.01^ab^	1.70	9.96^ab^	34.15	2.00	1.22^a^
	GLU	62.85^b^	0.33	11.05^b^	3.11	12.87^b^	1.57	10.98^a^	33.71	1.67	0.89^b^
	VIT	64.87^a^	0.33	15.29^a^	4.09	15.91^a^	1.63	8.61^b^	34.41	1.75	0.95^b^
	**SEM**	**1.14**	**0.01**	**1.01**	**0.46**	**0.81**	**0.14**	**0.57**	**0.22**	**0.22**	**0.05**
	***p*-value**	**0.03**	**0.46**	**0.01**	**0.31**	**0.03**	**0.79**	**0.02**	**0.09**	**0.54**	**<0.01**
Incubation Temperature (°C)	36.5	62.91	0.32	14.36	4.50	16.67^a^	2.01^a^	12.49^a^	34.01	1.84	0.99^ab^
	37.0	62.86	0.31	12.17	3.23	12.67^b^	1.08^b^	7.86^c^	34.00	1.57	1.19^a^
	37.5	60.98	0.34	11.83	3.29	14.40^ab^	1.81^c^	9.20^b^	34.27	2.01	0.89^b^
	**SEM**	**1.14**	**0.01**	**1.01**	**0.46**	**0.81**	**0.14**	**0.57**	**0.22**	**0.22**	**0.05**
	***p*-value**	**0.40**	**0.09**	**0.18**	**0.11**	**0.01**	**<0.01**	**<0.01**	**0.62**	**0.37**	**0.01**
	36.5										

Means that carry different superscripts are significantly different; **WRYS:** wet residual yolk sac; **DRYS**: dry residual yolk sac; **mm:** millimetre; **°C:** degree Celsius; **g:** gram**s; CON:** control; **GLU:** glucose: **VIT:** vitamin D3; **SEM:** standard error of means; ***p*-value**: probability value (*p* < 0.05).

**Table 2:** Effects of incubation temperature and *in ovo* feeding on embryonic development days 17 and 18.

Parameters	Feeding Group	Egg Weight (g)	Eggshell Thickness (mm)	Wet RYS (g)	Dry RYS (g)	Wet Embryo Weight (g)	Dry Embryo Wet (g)	Embryo Length (mm)	Embryo Temperature (°C)	Femur Length (mm)	Tibia Length (mm)
	INCUBATION DAY 17										
*In Ovo* Feeding (IOF)	CON	62.79	0.34	14.99	5.51	18.51^b^	2.71	23.93	33.76	2.37	1.36^ab^
	GLU	63.05	0.33	14.91	5.45	17.81^b^	2.70	23.82	33.90	2.19	1.23^b^
	VIT	61.73	0.33	23.27	5.17	21.11^a^	3.10	23.51	34.10	2.32	1.46^a^
	**SEM**	**1.36**	**0.01**	**5.79**	**0.38**	**0.63**	**0.18**	**0.83**	**0.20**	**0.07**	**0.05**
	***p*-value**	**0.77**	**0.53**	**0.51**	**0.79**	**0.02**	**0.21**	**0.93**	**0.49**	**0.20**	**0.01**
Temperature (Temp) (°C)	36.5	64.27	0.33^ab^	25.40	6.16^a^	21.33^a^	3.19^a^	24.42^a^	34.17	2.41	1.41^a^
	37.0	60.35	0.32^b^	12.69	4.37^b^	15.38^b^	2.05^b^	20.87^b^	33.58	1.97^b^	1.15^b^
	37.5	62.95	0.35^a^	15.10	5.60^ab^	20.72^a^	3.24^a^	25.60^a^	33.98	2.49	1.50^a^
	**SEM**	**1.36**	**0.01**	**5.79**	**0.38**	**0.63**	**0.18**	**0.83**	**0.20**	**0.07**	**0.05**
	***p*-value**	**0.13**	**0.01**	**0.27**	**0.01**	**<0.01**	**<0.01**	**<0.01**	**0.12**	**<0.01**	**<0.01**
	36.5										
*In Ovo* Feeding (IOF)	CON	61.71	0.33	14.99	5.83	23.93	3.82	12.57	34.38	2.53	1.36
	GLU	58.05	0.33	14.91	5.61	23.82	3.96	12.63	34.42	2.68	1.23
	VIT	60.89	0.31	23.27	5.53	23.51	3.65	12.15	34.02	2.43	1.46
	**SEM**	**1.15**	**0.01**	**5.79**	**0.37**	**0.83**	**0.18**	**0.24**	**0.19**	**0.07**	**0.04**
	***p*-value**	**0.07**	**0.23**	**0.51**	**0.84**	**0.93**	**0.45**	**0.32**	**0.25**	**0.05**	**0.49**
Incubation Temperature (°C)	36.5	63.23^a^	0.33	25.40	4.90^b^	24.42^a^	4.04^a^	12.80^a^	34.50^a^	2.62^a^	1.41^a^
	37.0	57.52^b^	0.33	12.69	6.51^a^	20.87^b^	3.32^b^	11.63^b^	33.65^b^	2.40^b^	1.15^b^
	37.5	59.89^ab^	0.31	15.10	5.60^ab^	25.60^a^	4.07^a^	12.95^a^	34.68^a^	2.70^a^	1.50^a^
	**SEM**	**1.15**	**0.01**	**5.79**	**0.37**	**0.83**	**0.18**	**0.24**	**0.19**	**0.07**	**0.04**
	***p*-value**	**0.01**	**0.09**	**0.27**	**0.01**	**<0.01**	**0.01**	**0.01**	**0.01**	**0.01**	**0.04**
	36.5										

Means that carry different superscripts are significantly different; **WRYS:** wet residual yolk sac; **DRYS**: dry residual yolk sac; **mm:** millimetre; **°C:** degree Celsius; **g:** gram**s; CON:** control; **GLU:** glucose: **VIT:** vitamin D3; **SEM:** standard error of means; ***p*-value**: probability value (*p* < 0.05).

**Table 3:** Effects of incubation temperature and *in ovo* feeding on embryonic development days 19 and 20.

Parameters	Feeding Group	Egg Weight (g)	Eggshell Thickness (mm)	Wet RYS (g)	Dry RYS (g)	Wet Embryo Weight (g)	Dry Embryo Weight (g)	Embryo Length (mm)	Embryo Temperature (°C)	Femur Length (mm)	Tibia Length (mm)
	INCUBATION DAY 19										
*In Ovo* Feeding (IOF)	CON	60.85	0.31	15.10	6.21^a^	28.10	5.38^a^	13.50	34.33	2.90	1.36
	GLU	61.44	0.31	14.03	5.70^b^	26.51	4.33^b^	13.31	34.10	2.90	1.23
	VIT	60.45	0.32	14.60	5.95^c^	25.93	4.37^b^	13.15	33.75	2.80	1.46
	**SEM**	**1.17**	**0.01**	**0.63**	**0.30**	**1.04**	**0.22**	**0.25**	**0.32**	**0.06**	**0.05**
	***p*-value**	**0.83**	**0.60**	**0.49**	**0.44**	**0.34**	**0.02**	**0.61**	**0.45**	**0.42**	**0.81**
Temperature (Temp) (°C)	36.5	63.74^a^	0.31	14.23	6.20^ab^	30.73^a^	5.23^a^	13.69^ab^	34.60^a^	3.02	1.41^ab^
	37.0	58.22^b^	0.30	14.57	5.17^b^	20.59^b^	3.97^b^	12.16^b^	32.90^b^	2.58	1.15^b^
	37.5	60.79^ab^	0.32	14.89	6.44^a^	29.19^ab^	4.90^a^	14.12^a^	34.70^a^	2.93	1.50^a^
	**SEM**	**1.17**	**0.01**	**0.63**	**0.30**	**1.04**	**0.22**	**0.25**	**0.32**	**0.07**	**0.06**
	***p*-value**	**0.01**	**0.21**	**0.76**	**0.01**	**<0.01**	**0.01**	**<0.01**	**<0.01**	**<0.01**	**<0.01**
	36.5										
*In Ovo* Feeding (IOF)	CON	62.80	0.32^a^	15.32^a^	6.23^a^	29.86^ab^	5.10	13.95	33.25	2.94	1.87
	GLU	61.11	0.29^b^	13.10^b^	5.19^ab^	31.20^a^	5.65	13.90	32.76	2.99	2.10
	VIT	60.54	0.29^b^	11.35^c^	4.75^b^	27.23^b^	5.20	13.97	32.80	2.93	1.97
	**SEM**	**1.06**	**0.01**	**0.62**	**0.39**	**0.91**	**0.19**	**0.25**	**0.18**	**0.08**	**0.07**
	***p*-value**	**0.32**	**0.04**	**<0.01**	**0.03**	**0.01**	**0.08**	**0.97**	**0.12**	**0.84**	**0.13**
Incubation Temperature (°C)	36.5	62.19	0.32^a^	13.25	5.50	32.98^a^	5.91^a^	14.61^a^	32.96	3.19^a^	2.04^ab^
	37.0	61.31	0.29^b^	13.37	5.10	23.59^b^	4.14^b^	12.82^b^	32.89	2.69^b^	1.81^b^
	37.5	60.91	0.29^b^	13.11	5.70	31.71^a^	5.90^a^	14.38^a^	32.97	2.97^b^	2.10^a^
	**SEM**	**1.06**	**0.01**	**0.62**	**0.39**	**0.91**	**0.19**	**0.25**	**0.18**	**0.08**	**0.07**
	***p*-value**	**0.68**	**0.02**	**0.96**	**0.55**	**<0.01**	**<0.01**	**<0.01**	**0.94**	**<0.01**	**0.02**
	36.5										

Means that carry different superscripts are significantly different; **WRYS:** wet residual yolk sac; **DRYS**: dry residual yolk sac; **mm:** millimetre; **°C:** degree Celsius; **g:** gram**s; CON:** control; **GLU:** glucose: **VIT:** vitamin D3; **SEM:** standard error of means; *p*-value: probability value (*p* < 0.05).

In addition, the results showed that the incubation temperature had a limited but significant effect on egg weight, with differences observed on ED18 and ED19 (*p* < 0.05). Thus, eggs incubated at 36.5°C consistently maintained higher weights compared with those incubated at 37.0°C and 37.5°C, while weight loss from ED15 to ED20 was greatest at 36.5°C (4.18%) and lowest at 37.5°C (2.77%) (Table 1, Table 2, Table 3).

Furthermore, the eggshell thickness was generally not influenced (*p* > 0.05) by *in ovo* feeding, except on ED20 (*p* < 0.05), where control eggs exhibited the greatest thickness (0.32 mm) compared with injected groups (0.29 mm). Similarly, it was observed that the incubation temperature affected shell thickness only on ED17 and ED20 (*p* < 0.05), with reduced thickness observed at higher temperatures (37.0°C and 37.5°C) relative to 36.5°C (Table 1, Table 2, Table 3).

### Residual yolk sac utilization

#### Wet residual yolk sac (RYS)

The results showed that the wet RYS weight was affected by *in ovo* feeding on ED16 and ED20 (*p* < 0.05) with control eggs consistently exhibiting higher values than vitamin D_3_ and glucose treatments. Across incubation, wet RYS weight increased slightly from ED15 to ED18, followed by a decline toward hatch, reflecting progressive yolk absorption. Incubation temperature influenced wet RYS weight only on ED15 (*p* < 0.05), where embryos incubated at 36.5°C showed higher values than those at 37.0 °C, with 37.5 °C intermediate (Table 1, Table 2, Table 3).

#### Dry residual yolk sac

Dry RYS weight was significantly affected by *in ovo* feeding on ED19 and ED20 (*p* < 0.05), with control eggs showing consistently higher values, followed by glucose and vitamin D₃ groups. However, incubation temperature significantly influenced dry RYS weight on ED17- ED19 (*p* < 0.05), where embryos incubated at 36.5°C consistently exhibited higher dry yolk reserves than those at 37.5°C and 37.0°C. Thus, dry RYS weight increased progressively with embryonic age across all treatments, indicating gradual lipid mobilization during development (Table 1, Table 2, Table 3).

### Embryonic growth performance

#### Embryo length

Embryo length was not significantly affected by *in ovo* feeding across most incubation days, except on ED16 and ED17 (*p* < 0.05), where control embryos were generally longer than glucose and vitamin D_3_ groups. Regardless of treatment, embryo length increased steadily with incubation age (*p* < 0.05). The incubation temperature had a consistent effect on embryo length (*p* < 0.05), with embryos incubated at 36.5°C showing the greatest length, followed by those at 37.5°C, while 37.0°C resulted in the shortest embryos (Table 1, Table 2, Table 3).

##### Wet embryo weight

The wet embryo weight was significantly influenced by *in ovo* feeding on ED16, ED17, and ED20 (*p* < 0.05), with control embryos generally exhibiting higher weights than glucose and vitamin D₃ treatments. In all groups, the wet embryo weight increased progressively throughout incubation. Incubation temperature significantly affected wet embryo weight on all incubation days (*p* < 0.05). The embryos incubated at 36.5°C consistently showed the highest weights, followed by 37.5°C, while 37.0°C produced the lowest values (Table 1, Table 2, Table 3).

##### Dry embryo weight

Dry embryo weight was influenced by *in ovo* feeding only on ED19 (*p* < 0.05), where control embryos (5.38 g) were significantly heavier than vitamin D₃ (4.37 g) and glucose (4.33 g) groups. No other differences were observed across earlier stages. Incubation temperature significantly influenced dry embryo weight on all days except ED15 (*p* < 0.05), with 36.5°C producing the highest values, followed by 37.5 °C, with 37.0 °C consistently resulting in the lowest embryo dry mass (Tables 1–3).

#### Embryo temperature

*In ovo* feeding affected embryo temperature only on ED15 (*p* < 0.05), with no consistent treatment differences thereafter. Embryo temperature showed a slight decline as incubation progressed. The incubation temperature significantly influenced embryo temperature on ED15, ED18, and ED19 (*p* < 0.05). The eggs incubated at 37.5°C generally exhibited the highest embryo temperatures, followed by 36.5°C, while 37.0°C remained the lowest (Table 1, Table 2, Table 3).

### Skeletal development

#### Femur length

The femur length was not affected by *in ovo* feeding (*p* > 0.05), but increased progressively with embryonic age in all treatments. The incubation temperature significantly influenced femur length on ED15, ED17, ED18, and ED20 (*p* < 0.05), with the greatest values observed at 37.5°C, intermediate at 36.5°C, and lowest at 37.0°C (Table 1, Table 2, Table 3).

#### Tibia length

Tibia length was influenced by *in ovo* feeding during early development (ED15 to ED17) (*p* < 0.05), with vitamin D₃-injected embryos showing the greatest tibia length, followed by control and glucose groups. From ED18 onward, no significant differences were observed. Incubation temperature consistently affected tibia length throughout theincubation period (*p* < 0.05), with the highest values recorded at 37.5°C, intermediate at 36.5°C, and lowest at 37.0°C (Table 1, Table 2, Table 3).

#### Interaction effects (*in ovo* feeding × incubation temperature)

Significant interactions between *in ovo* feeding and incubation temperature were observed for multiple traits, including egg weight (ED18), wet RYS weight (ED19), dry RYS weight (ED17-ED19), embryo length (all days), wet embryo weight (most days except ED16), dry embryo weight (all days), embryo temperature (ED15, ED17-ED19) (*p* < 0.05), femur length (ED15 and ED17) (*p* < 0.05), and tibia length (ED16, ED17, and ED19) (*p* < 0.05) (Table 4). Thus, embryos from vitamin D_3_-injected eggs incubated at 36.5°C frequently exhibited enhanced skeletal development and growth performance, whereas embryos incubated at 37.0°C consistently showed reduced developmental indices. However, interaction effects were not uniform across traits or developmental stages, indicating that responses were both stage-dependent and trait-specific rather than universally synergistic (Table 4).

**Table 4:** Interactive effect of early *in ovo* feeding and incubation temperature on the quality of broiler eggs during incubation.

Incubation Day	*In Ovo* Feed X Incub Temp	Egg Weight (g)	EST (mm)	Wet RYS (g)	Dry RYS (g)
**15**	CON*36.5°C	67.24	0.34	15.32	4.36
	CON*37.0°C	64.82	0.32	8.90	2.68
	CON*37.5°C	62.78	0.33	12.44	4.42
	GLU*36.5°C	61.54	0.33	13.48	3.98
	GLU*37.0°C	62.44	0.33	7.88	3.66
	GLU*37.5°C	64.28	0.33	12.06	2.66
	VIT*36.5°C	65.90	0.31	12.34	3.24
	VIT*37.0°C	63.34	0.32	8.88	2.74
	VIT*37.5°C	61.48	0.32	12.74	3.26
**SEM**		**1.58**	**0.91**	**1.69**	**0.76**
***p*-value**		**0.17**	**0.03**	**0.88**	**0.48**
**16**	CON*36.5°C	59.56	0.31	13.32	4.84
	CON*37.0°C	59.74	0.31	10.42	3.10
	CON*37.5°C	57.80	0.33	12.36	3.38
	GLU*36.5°C	64.42	0.32	13.46	3.44
	GLU*37.0°C	63.64	0.33	10.00	3.08
	GLU*37.5°C	60.48	0.35	9.67	2.80
	VIT*36.5°C	64.74	0.33	16.30	5.10
	VIT*37.0°C	65.20	0.30	16.10	3.50
	VIT*37.5°C	64.68	0.35	13.46	3.68
**SEM**		**1.97**	**1.40**	**1.75**	**0.79**
***p*-value**		**0.90**	**0.49**	**0.69**	**0.92**
**17**	CON*36.5°C	66.26	0.33	17.66	6.60^ab^
	CON*37.0°C	60.30	0.34	19.84	3.62^c^
	CON*37.5°C	61.82	0.35	17.46	6.30^ab^
	GLU*36.5°C	61.90	0.33	13.46	4.78^b^
	GLU*37.0°C	61.82	0.31	15.80	5.24^b^
	GLU*37.5°C	65.42	0.35	15.48	6.34^ab^
	VIT*36.5°C	64.64	0.32	17.08	7.10^a^
	VIT*37.0°C	58.92	0.30	12.42	4.26^b^
	VIT*37.5°C	61.62	0.36	12.32	4.14^b^
**SEM**		**2.35**	**1.09**	**10.0**	**0.66**
***p*-value**		**0.47**	**0.02**	**0.33**	**0.01**
**18**	CON*36.5°C	64.96^a^	0.34	17.66	3.26^c^
	CON*37.0°C	59.26^ab^	0.31	19.84	7.30^a^
	CON*37.5°C	60.90^ab^	0.29	17.46	6.94^ab^
	GLU*36.5°C	58.62^ab^	0.32	13.46	5.52^b^
	GLU*37.0°C	55.56^b^	0.32	15.80	5.50^b^
	GLU*37.5°C	59.98^ab^	0.33	15.48	5.82^b^
	VIT*36.5°C	66.12^a^	0.33	17.08	5.86^b^
	VIT*37.0°C	57.74^ab^	0.35	12.42	6.74^ab^
	VIT*37.5°C	58.80^ab^	0.31	12.32	3.98^bc^
**SEM**		**1.98**	**1.44**	**10.0**	**0.65**
***p*-value**		**0.04**	**0.04**	**0.33**	**0.01**
**19**	CON*36.5°C	62.18	0.31	15.94^a^	7.52^a^
	CON*37.0°C	56.98	0.30	13.56^b^	4.10^c^
	CON*37.5°C	63.40	0.32	15.80^a^	7.02^ab^
	GLU*36.5°C	65.06	0.33	11.42^c^	5.06^bc^
	GLU*37.0°C	58.96	0.30	15.36^a^	5.16^bc^
	GLU*37.5°C	60.32	0.30	15.32^a^	6.74^b^
	VIT*36.5°C	63.98	0.30	15.34^a^	6.02^b^
	VIT*37.0°C	58.72	0.31	14.80^ab^	6.26^b^
	VIT*37.5°C	58.64	0.34	13.54^b^	5.56^bc^
**SEM**		**2.03**	**1.81**	**1.08**	**0.53**
***p*-value**		**0.41**	**0.04**	**0.03**	**0.01**
**20**	CON*36.5°C	64.74	0.35	14.82	5.40
	CON*37.0°C	60.72	0.29	15.56	6.10
	CON*37.5°C	62.80	0.33	15.58	7.18
	GLU*36.5°C	60.70	0.32	13.82	5.44
	GLU*37.0°C	61.52	0.29	12.94	4.94
	GLU*37.5°C	61.12	0.28	12.44	5.20
	VIT*36.5°C	61.12	0.31	11.12	5.54
	VIT*37.0°C	61.70	0.29	11.60	4.14
	VIT*37.5°C	58.80	0.28	11.32	4.58
**SEM**		**1.83**	**1.58**	**1.07**	**0.44**
***p*-value**		**0.54**	**0.01**	**0.88**	**0.02**

Means that carry different superscripts are significantly different; **EW:** egg weight; **EST:** eggshell thickness; **mm:** millimetre; **WRYS:** wet residual yolk sac; **DRYS**: dry residual yolk sac; **g:** gram**s; CON:** control; **GLU:** glucose: **VIT:** vitamin; **°C:** degree Celsius; **IT:** incubation temperature; **SEM:** standard error of means; *p*-value: probability value (*p* < 0.05).

#### Interactive effects of early *in ovo* feeding and incubation temperature on embryonic development during incubation

Significant *in ovo* feeding × incubation temperature interactions (*p* < 0.05) were observed for embryo length, wet and dry embryo weights, embryo temperature, femur length, and tibia length (Table 5). Across the incubation days, vitamin D_3_ supplementation combined with 36.5°C incubation consistently produced the highest embryo growth and skeletal measurements, while incubation at 37.0°C generally resulted in the lowest values (Table 5).

**Table 5:** Interactive effect of early *in ovo* feeding and incubation temperature on embryonic development during incubation.

Incubation Day	IOF X IT	EL (mm)	Wet Embryo Weight (g)	Dry Embryo Weight (g)	ET (°C)	FL (mm)	TL (mm)
**15**	CON*36.5°C	8.80^ab^	15.62^a^	1.44^a^	36.90^a^	1.24^ab^	1.08
	CON*37.0°C	9.10^a^	10.46^bc^	0.82^a^	33.58^b^	0.80^bc^	0.66
	CON*37.5°C	8.98^ab^	13.72^ab^	1.48^a^	35.10^ab^	1.14^abc^	0.86
	GLU*36.5°C	9.22^a^	12.62^abc^	1.26^ab^	34.38^bc^	1.12^ab^	0.78
	GLU*37.0°C	7.88^b^	8.70^c^	0.64^b^	33.04^c^	0.68^c^	0.48
	GLU*37.5°C	8.90^ab^	13.66^ab^	1.12^ab^	34.78^bc^	1.00^ab^	0.68
	VIT*36.5°C	9.40^a^	13.76^ab^	1.40^a^	33.34^b^	1.38^a^	0.92
	VIT*37.0°C	8.18^b^	10.88^bc^	0.92^ab^	34.08^b^	0.94^ab^	0.82
	VIT*37.5°C	8.92ab	13.72^ab^	1.38^a^	34.38^bc^	0.82^bc^	0.72
**SEM**		**0.29**	**0.91**	**0.16**	**0.41**	**0.11**	**0.09**
***p*-value**		**0.03**	**0.03**	**0.05**	**0.01**	**0.01**	**0.05**
**16**	CON*36.5°C	10.72^b^	16.74	2.08^ab^	33.50	1.64	0.78^d^
	CON*37.0°C	8.86^bc^	12.16	1.14^ab^	34.70	1.64	1.64^a^
	CON*37.5°C	10.30^b^	16.12	1.88^ab^	34.26	2.72	1.24^ab^
	GLU*36.5°C	16.38^a^	15.28	1.76^ab^	34.30	1.80	0.96^bcd^
	GLU*37.0°C	7.84^bc^	10.46	0.96^b^	33.06	1.56	0.92^bcd^
	GLU*37.5°C	8.72^bc^	12.86	1.98^ab^	33.78	1.64	0.80^cd^
	VIT*36.5°C	10.36^b^	18.0	2.18^a^	34.22	2.08	1.32^abc^
	VIT*37.0°C	6.88^c^	15.38	1.14^ab^	34.24	1.52	1.00^bcd^
	VIT*37.5°C	8.58^bc^	14.34	1.56^ab^	34.78	1.66	0.64^d^
**SEM**		**0.98**	**1.40**	**0.24**	**0.39**	**0.38**	**0.09**
***p*-value**		**0.04**	**0.49**	**0.54**	**0.05**	**0.32**	**0.01**
**17**	CON*36.5°C	11.42^abc^	21.60^b^	3.12^abc^	33.26^c^	2.36^abc^	1.44^abcd^
	CON*37.0°C	8.58^d^	12.70^d^	1.74^c^	33.72^bc^	2.02^c^	1.08^d^
	CON*37.5°C	12.12^a^	21.24^b^	3.26^ab^	34.30^a^	2.72^ab^	1.56^ab^
	GLU*36.5°C	9.76^bcd^	18.28^bc^	2.66^abc^	34.46^a^	2.14^bc^	1.16^cd^
	GLU*37.0°C	9.58^cd^	14.96^cd^	2.04^bc^	33.54^bc^	1.92^c^	1.16^cd^
	GLU*37.5°C	11.54^ab^	20.18^b^	3.38^ab^	33.62^bc^	2.50^abc^	1.38^abcd^
	VIT*36.5°C	12.30^a^	24.10^a^	3.80^a^	34.80^a^	2.74^a^	1.64^a^
	VIT*37.0°C	10.04^bcd^	18.48^bc^	2.38^abc^	33.48^bc^	1.98^c^	1.20^bcd^
	VIT*37.5°C	12.06^a^	20.74^b^	3.08^abc^	34.02^a^	2.24^abc^	1.54^abc^
**SEM**		**0.42**	**1.09**	**0.31**	**0.35**	**0.13**	**0.08**
***p*-value**		**0.01**	**0.02**	**0.02**	**0.04**	**0.01**	**0.06**
**18**	CON*36.5°C	12.36^b^	23.76^ab^	4.06^ab^	34.20^ab^	2.48	1.68
	CON*37.0°C	11.34^bc^	19.74^c^	2.82^c^	33.14^c^	2.32	1.58
	CON*37.5°C	14.00^a^	28.30^a^	4.58^a^	35.82^a^	2.80	1.80
	GLU*36.5°C	13.26^ab^	24.08^ab^	4.16^ab^	34.76^ab^	2.74	1.80
	GLU*37.0°C	11.24^c^	19.92^bc^	3.28^bc^	34.38^abc^	2.44	1.60
	GLU*37.5°C	13.40^a^	26.54^ab^	4.44^a^	34.12^bc^	2.86	1.68
	VIT*36.5°C	12.68^b^	25.42^ab^	3.90^ab^	34.54^ab^	2.64	1.66
	VIT*37.0°C	12.32^b^	22.94^b^	3.86^bc^	33.42^c^	2.34	1.58
	VIT*37.5°C	11.46^bc^	23.10^b^	3.18^bc^	34.10^abc^	2.32	1.66
**SEM**		**0.41**	**1.44**	**0.32**	**0.32**	**0.12**	**0.08**
***p*-value**		**0.01**	**0.04**	**0.01**	**0.00**	**0.09**	**0.05**
**19**	CON*36.5°C	13.12^abc^	28.88^ab^	5.06^b^	35.24^ab^	2.94	1.76^abc^
	CON*37.0°C	13.08^abc^	24.74^ab^	6.04^a^	32.62^c^	2.70	1.82^abc^
	CON*37.5°C	14.30^a^	30.60^a^	5.06^b^	35.12^ab^	3.06	1.20^ab^
	GLU*36.5°C	13.96^a^	31.40^a^	5.16^b^	33.82^b^	3.04	1.94^abc^
	GLU*37.0°C	11.60^c^	19.02^b^	2.78^c^	32.78^bc^	2.50	1.52^c^
	GLU*37.5°C	14.38^a^	29.10^ab^	5.04^bc^	35.70^a^	3.02	2.04^ab^
	VIT*36.5°C	13.98^a^	31.92^a^	5.48^b^	34.60^ab^	3.08	1.64^a^
	VIT*37.0°C	11.80^bc^	18.02^b^	3.10^b^	33.24^b^	2.54	1.60^bc^
	VIT*37.5°C	13.68^ab^	27.86^ab^	4.54^bc^	33.40^b^	2.70	1.88^abc^
**SEM**		**0.43**	**1.81**	**0.38**	**0.56**	**0.12**	**0.10**
***p*-value**		**0.02**	**0.04**	**0.01**	**0.04**	**0.21**	**0.01**
**20**	CON*36.5°C	14.24^ab^	31.42^ab^	5.50^abc^	33.68	3.34	1.92
	CON*37.0°C	12.64^c^	22.87^c^	6.25^ab^	33.20	2.48	1.70
	CON*37.5°C	14.98^a^	35.30^a^	6.06^ab^	32.88	3.00	2.00
	GLU*36.5°C	14.70^ab^	34.40^ab^	6.06^ab^	32.58	3.20	2.20
	GLU*37.0°C	12.86^bc^	24.92^bc^	4.14^cd^	32.80	2.78	1.92
	GLU*37.5°C	14.10^ab^	34.22^ab^	6.74^a^	32.90	2.98	2.14
	VIT*36.5°C	14.90^a^	33.10^b^	6.16^ab^	32.62	3.04	2.00
	VIT*37.0°C	12.96^bc^	22.98^c^	4.66^bcd^	32.66	2.80	1.80
	VIT*37.5°C	14.06^abc^	25.64^bc^	4.78^bcd^	33.12	2.94	2.10
**SEM**		**0.44**	**1.58**	**0.33**	**0.32**	**0.13**	**0.13**
***p*-value**		**0.02**	**0.01**	**0.02**	**0.29**	**0.201**	**0.97**

Means that carry different superscripts are significantly different; **EL:** embryo length; **WEW:** wet embryo weight; **DEW:** dry embryo weight; **ET:** embryo temperature**; FL:** femur length; **TL**: tibia length; **mm:** millimetre; **°C:** degree Celsius; **g:** gram**s; CON:** control; **GLU:** glucose: **VIT:** vitamin D3; **IT:** incubation temperature; **IOF:***in ovo* feeding; **SEM:** standard error of means; *p*-value: probability value (*p* < 0.05).

## Discussion

The present study revealed that egg weight declined progressively across incubation days in all treatments, reflecting normal water loss through the shell and progressive utilization of egg nutrients for embryonic growth and metabolism (Alsobayel and Albadry, 2011; Jin et al., 2011; Kruenti et al., 2022). *In ovo* feeding exerted limited influence on egg weight, with significant differences detected only on specific days, specifically day 16 and, under interaction analysis, day 18. The transiently greater egg weight observed in vitamin D_3_-injected eggs on day 16 suggests short-term alterations in nutrient mobilization or water dynamics; however, across most incubation days, control eggs maintained slightly higher weights than supplemented groups, indicating relatively greater nutrient utilization in injected eggs (Maman et al., 2019). Incubation temperature affected egg weight primarily toward the end of incubation (days 18 and 19), with eggs incubated at 36.5°C generally retaining higher weights than those incubated at 37.0 or 37.5°C. The lower egg weight observed at 37.0°C on these days suggests enhanced metabolic rate and nutrient turnover at this temperature, consistent with the temperature-dependent modulation of embryonic metabolism reported by Maatjens et al. (2014), Narinç et al., 2016, Lourens et al., 2007, and Leksrisompong et al. (2007).

Eggshell thickness was largely unaffected by *in ovo* supplementation or incubation temperature, except on isolated days (specifically days 17 and 20). The marginal reduction in shell thickness toward day 20, particularly at higher temperatures, is consistent with progressive mobilization of shell calcium to support skeletal mineralization (de Juan et al., 2023). Although vitamin D_3_ is central to calcium and phosphorus metabolism, its *in ovo* administration did not significantly alter shell thinning patterns, suggesting that calcium resorption remained primarily governed by developmental stage rather than treatment (Sun et al., 2025).

The residual yolk sac (RYS) dynamics demonstrated that the wet RYS weight was influenced by *in ovo* feeding on days 16 and 20 and by incubation temperature mainly on day 15. In addition, the lower wet RYS weight in glucose-injected eggs on specific days indicates enhanced yolk absorption at particular developmental stages (Miri et al., 2022; Thamteerasathian et al., 2026). The temperature-related reductions in RYS weight, especially at 37.0 °C on selected days, further suggest increased metabolic activity and nutrient utilization at moderate incubation temperature, whereas, the dry RYS weight was more responsive to treatment interactions, with significant effects observed on days 17 to 19. Toward the end of the incubation, supplemented eggs often exhibited lower dry RYS weights than controls, indicating greater transfer of yolk nutrients to embryonic tissues. These findings align with previous observations that incubation temperature modulates yolk utilization efficiency and chick quality (Hamidu et al., 2011; Willemsen et al., 2010; Willemsen et al., 2011; Nangsuay et al., 2013). Thus, the results confirm that both nutrient supplementation and thermal environment influence the timing and extent of yolk sac absorption, although effects are not uniform throughout incubation.

The embryo length increased consistently with advancing incubation age, however, the *in ovo* feeding affected the embryo length only on days 16 and 17, with control embryos generally longer than those from supplemented eggs under main-effect comparisons. Whereas, significant feeding × temperature interactions indicate that embryonic linear growth responds to combined nutritional and thermal conditions. For example, the embryo length was enhanced at 37.5°C in the control eggs during the late incubation, whereas vitamin D_3_-injected eggs demonstrated improved length at 36.5°C (Lin et al., 2017; Tona et al., 2022). The wet embryo weight increased steadily with incubation age and was significantly influenced by both feeding and temperature. Although control eggs frequently exhibited higher wet embryo weight in main-effect analysis, interaction results revealed that vitamin D_3_ supplementation at 36.5°C yielded some of the highest embryo weights across incubation days. Dry embryo weight followed similar patterns, increasing progressively and showing significant temperature effects on most days. These findings suggest that moderate incubation temperature may provide optimal metabolic conditions for embryos receiving exogenous vitamin D_3_, potentially enhancing nutrient assimilation and tissue deposition (Yalcin et al., 2022).

The embryo temperature showed a slight decreasing trend as incubation progressed, whereas, the treatment effects were intermittent, and embryos from control eggs generally exhibited marginally higher temperatures than those from the supplemented eggs. The absence of a strictly linear relationship between incubator set temperature and measured embryo temperature supports previous findings by Agyekum et al. (2022), who emphasized that embryonic heat production and eggshell conductance can decouple embryo temperature from incubator settings.

The skeletal development parameters responded differentially to treatments. Thus, the femur length increased consistently with incubation age and was influenced primarily by incubation temperature rather than feeding, except on selected days. The tibia length was more responsive to *in ovo* feeding during mid-incubation (days 15-17), with vitamin D_3_-injected eggs often exhibiting greater tibial length. Specifically, the combination of vitamin D_3_ supplementation and 36.5°C incubation consistently supported superior femur and tibia development. Given the established role of vitamin D₃ in regulating calcium metabolism and bone mineralization, these findings are biologically plausible and align with reports of enhanced skeletal traits following prenatal nutrient supplementation (Abbasi et al., 2017; Imran et al., 2023). The moderate incubation temperature of 36.5°C may have optimized mineral mobilization from the shell without imposing thermal stress, thereby supporting efficient bone elongation.

Across variables, responses were not uniformly linear with increasing incubation temperature. Thus, while 37.0°C promoted greater nutrient utilization on certain days, as reflected by reduced egg and yolk weights, 36.5°C more consistently supported embryonic growth and skeletal development when combined with vitamin D_3_ supplementation. The incubation temperature at 37.5°C occasionally enhanced linear growth but did not consistently optimize embryo weight or bone metrics (Yalcin et al., 2022). In addition, the superior embryo growth and skeletal development observed in vitamin D_3_-supplemented eggs incubated at 36.5°C suggest that moderate incubation temperature may enhance the utilization of vitamin D_3_ for tissue accretion and bone elongation. However, the reduced growth responses at 37.0°C indicate that embryonic developmental outcomes are highly dependent on the interaction between nutrient supplementation and the thermal environment. These results underscore the importance of achieving a balance between metabolic rate and nutrient availability during embryogenesis. Hence, excessively high or suboptimal temperatures may alter metabolic efficiency and tissue accretion patterns, even when supplemental nutrients are provided.

## Conclusion

Taken together, the results obtained from this study showed that *in ovo* administration of glucose or vitamin D_3_ and variation in constant incubation temperature significantly influenced specific egg quality and embryonic developmental traits, with effects dependent on incubation stage and trait measured. Although glucose supplementation enhanced yolk utilization on selected days, vitamin D₃ supplementation combined with incubation at 36.5°C most consistently improved embryonic growth performance, particularly embryo weight and skeletal development. Thus, the incubation at 37.0°C promoted nutrient utilization at certain stages but did not uniformly optimize embryonic growth. The interaction between *in ovo* nutrient supplementation and incubation temperature significantly influenced embryonic growth and skeletal development. Under the conditions of this study, vitamin D_3_ administration combined with incubation at 36.5°C produced the most favorable embryonic developmental profile, characterized by enhanced embryo growth and bone development. Further studies should evaluate the post-hatch growth and physiological consequences of these prenatal interventions to determine their long-term production relevance.

## Funding statement

The study was supported by grants from the China-Togo Joint Laboratory on Poultry Nutrition and Intestinal Health (2025YFE0124600); the Earmarked Fund for China Agriculture Research System under Grant number CARS-40-K09; and the University Youth Innovation Team of Shandong Province under Grant number 2024KJI005.

## CRediT authorship contribution statement

**Maxwell Ansong Okai:** Writing – review & editing, Writing – original draft, Project administration, Methodology, Investigation, Formal analysis, Data curation, Conceptualization. **Francis Kruenti:** Writing – review & editing, Writing – original draft, Formal analysis. **Yendouhamtchié Nadiedjoa:** Writing – review & editing, Writing – original draft, Methodology, Data curation. **Achiamaa Asafu-Adaye Koranteng:** Writing – review & editing, Writing – original draft, Formal analysis. **Vida Korkor Lamptey:** Writing – review & editing, Writing – original draft, Formal analysis. **Benjamin. Adjei-Mensah:** Writing – review & editing, Writing – original draft, Software, Formal analysis. **Jacob Alhassan Hamidu:** Writing – review & editing, Writing – original draft, Methodology, Formal analysis, Conceptualization. **Kokou Tona:** Writing – review & editing, Writing – original draft, Supervision, Methodology. **Felix Kwame Amevor:** Writing – review & editing, Writing – original draft, Software, Methodology. **Hai Lin:** Writing – review & editing, Writing – original draft, Validation, Supervision, Resources, Project administration, Methodology, Investigation, Funding acquisition, Conceptualization.

## Disclosures

The authors state that they have no financial or non-financial conflicts of interest in submitting this manuscript.

## References

[bib0001] Abbasi T., Shakeri M., Zaghari M., Kohram H. (2017). Growth performance parameters, bone calcification and immune response of *in ovo* injection of 25 hydroxycholecalciferol and vitamin broilers. Theriogenology.

[bib0002] Abuoghaba A.A. (2017). Impact of spraying incubated eggs submitted to high temperature with ascorbic acid on embryonic development, hatchability, and some physiological responses of hatched chicks. Can. J. Anim. Sci..

[bib0004] Alsobayel A.A., Albadry M.A. (2011). Effect of storage period and strain of layer on internal and external quality characteristics of eggs marketed in Riyadh area. J. Saudi Societ. Agric. Sci..

[bib0005] Bello A., Zhai W., Gerard P.D., Peebles E.D. (2014). Effects of the commercial *in ovo* injection of 25-hydroxycholecalciferol on the hatchability and hatching chick quality of broilers. Poult. Sci..

[bib0006] Browne T.J. (2006). Wild Life Management Report (No. 197A).

[bib0007] Dayan J., Reicher N., Melkman-Zehavi T., Uni Z. (2020). Incubation temperature affects yolk utilisation through changes in the expression of yolk sac tissue functional genes. Poult. Sci..

[bib0008] Decuypere E., Michels H. (1992). Incubation temperature as a management tool: a review. World’s Poult. Sci. J..

[bib0009] de Juan A.F., Scappaticcio R., Aguirre L., Fondevila G., García J., Cámara L., Mateos G.G. (2023). Influence of the calcium and nutrient content of the prelay diet on egg production, egg quality, and tibiae mineralization of brown egg-laying hens from 16 to 63 wk of age. Poult. Sci..

[bib0010] Dong X.Y., Jiang Y.J., Wang M.Q., Wang Y.M., Zou X.T. (2013). Effects of *in ovo* feeding of carbohydrates on hatchability, body weight, and energy status in domestic pigeons (*Columba livia*). Poult. Sci..

[bib0011] French N.A. (1997). Modeling incubator temperature: the effects of incubator design, embryonic development, and egg size. Poult. Sci..

[bib0012] Gualhanone A., Furlan R.L., Fernandez-Alarcon M.F., Macari M. (2012). Effect of breeder age on eggshell thickness, surface temperature, hatchability and chick weight. Braz. J. Poult. Sci..

[bib0013] Hamidu J.A., Fasenko G.M., Guan L., Barreda D.R. (2011). Influence of parent flock age on embryonic metabolism in modern turkey strains. Poult. Sci..

[bib0015] Imran A., Nasir R., Imdad H.L., Nasir M., Zahid I.R. (2023). In-ovo mineral supplementation: a comprehensive analysis of broiler bone morphometry and structural implications. Pakist. J. Multidiscip. Res..

[bib0003] Agyekum G., Okai M.A., Tona J.K., Donkoh A., Hamidu A. (2022). Impact of incubation temperature profile on chick quality, bone, and immune system during the late period of incubation of Cobb 500 broiler strain. Poult. Sci..

[bib0016] Jin Y.H., Lee K.T., Lee W.I., Han Y.K. (2011). Effects of storage temperature and time on the quality of eggs from laying hens at peak production. Asian-Aust. J. Anim. Sci..

[bib0017] Kruenti F., Hagan K.J., Okai M.A., Lamptey K.V. (2022). The quality of white and brown chicken eggs kept under different storage length and storage temperatures. J. Innov. Agric..

[bib0019] Leksrisompong N., Romero-Sanchez H., Plumstead P.W., Brannan K.E., Brake J. (2007). Broiler incubation.1. Effect of elevated temperature during late incubation on body weight and organs of chicks. Poult. Sci..

[bib0020] Lin Y.M., Druyan S., Yahav S., Brake J. (2017). Thermal treatments prior to and during the beginning of incubation affects development of the broiler embryo and yolk sac membranes, and live performance and carcass characteristics. Poult. Sci..

[bib0022] Lourens A., Brand H.V.D., Heetkamp M.J.W., Meijerhof R., Kemp B. (2007). Effects of eggshell temperature and oxygen concentration on embryo growth and metabolism during incubation. Poult. Sci..

[bib0023] Maatjens C.M., Reijrink I.A.M., Molenaar R., van der Pol C.W., Kemp B., van den Brand H. (2014). Temperature and CO2 during the hatching phase. I. Effects on chick quality and organ development. Poult. Sci..

[bib0024] Maatjens C.M., van Roovert-Reijrink I.A.M., Engel B., van der Pol C.W., Kemp B., van den Brand H. (2016). Temperature during the last week of incubation. I. Effects on hatching pattern and broiler chicken embryonic organ development. Poult. Sci..

[bib0025] Maman A.H., Aygün A., Yıldırım İ., Alsadoon M.K.K (2019). Effects of in-ovo injection of D3 vitamin on hatchability and supply organ weights in quail hatching eggs. Bahri Dağdaş Hayvancılık Araştırma Dergisi.

[bib0026] Masia K.S., Idowu P.A., Nephawe K.A., Mtileni B., Ngcobo J.N., Modiba M.C., Mpofu T.J. (2025). Effect of incubation temperature on hatchability, chick quality and post-hatch performance-review. Anim. Sci. Genet..

[bib0027] Miri B., Ghasemi A.H., Hajkhodadadi I., Farahani A.H.K. (2022). Effects of low eggshell temperatures during incubation, *in ovo* feeding of l-arginine, and post-hatch dietary guanidinoacetic acid on hatching traits, performance, and physiological responses of broilers reared at low ambient temperature. Poult. Sci..

[bib0028] Moura L., Vakharia V., Liu M., Song H. (2007). *In ovo* vaccine against infectious bursal disease. Int. J. Poult. Sci..

[bib0029] Nangsuay A., Meijerhof R., Ruangpanit Y., Kemp B., van den Brand H. (2013). Energy utilization and heat production of embryos from eggs originating from young and old broiler breeder flocks. Poult. Sci..

[bib0030] Narinç D., Erdoğan S., Tahtabiçen E., Aksoy T. (2016). Effects of thermal manipulations during embryogenesis of broiler chickens on developmental stability, hatchability and chick quality. Animals.

[bib0031] Peebles E.D. (2018). *In ovo* applications in poultry: a review. Poult. Sci..

[bib0032] Peebles E.D., Barbosa T.M., Cummings T.S., Dickson J., Womack S.K., Gerard P.D. (2017). Comparative effects of *in ovo* versus subcutaneous administration of the Marek’s disease vaccine and pre-placement holding time on the processing yield of Ross 708 broilers. Poult. Sci..

[bib0033] Peebles E.D., Barbosa T.M., Cummings T.S., Dickson J., Womack S.K. (2016). Comparative effects of *in ovo* versus subcutaneous administration of the Marek’s disease vaccine and pre-placement holding time on the early post-hatch quality of Ross × Ross 708 broiler chicks. Poult. Sci..

[bib0034] Retes A.M., Oliveira C.A. (2016). Carbohydrate injection and its effects on energy utilization in chicken embryos. Poult. Sci..

[bib0035] Sgavioli S., Santos E.T., Domingues C.H.F., Quadros T.C.O., Castiblanco D.M.C., Andrade-Garcia G.M., Amoroso L., Nääs I.A., Garcia R.G., Baraldi-Artoni S.M. (2016). Effect of high incubation temperature on the blood parameters of layer chicks. Rev. Bras. Cienc. Avic..

[bib0036] Sokale A.O., Williams C.J., Cummings T.S., Gerard P.D., Bello A., Peebles E.D. (2018). Effects of *in ovo* injection of different doses of coccidiosis vaccine and turn-out times on broiler performance. Poult. Sci..

[bib0037] Speier J.S., Yadgary L., Uni Z., Wong E.A. (2012). Gene expression of nutrient transporters and digestive enzymes in the yolk sac membrane and small intestine of the developing embryonic chick. Poult. Sci..

[bib0038] Sun Y., Zhang G., Zhao B., Fu C., Li B., Wang F., Tian M., Wang M., Lin H., Li H. (2025). *In ovo* vitamin D3 injection leads to a long-term improvement of bone development in laying chicks. Poult. Sci.

[bib0041] Thamteerasathian L., Chaiworakul V., Poeikhampha T., Kulsing C., Tungkijanansin N., Anal A.K. (2026). Vitamin D enrichment of eggs and its effects on performance and intestinal health in late-stage laying hens. Poult. Sci..

[bib0042] Tona K., Voemesse K., N'nanlé O., Oke O.E., Kouame Y.A.E., Bilalissi A., Meteyake H., Oso O.M (2022). Chicken incubation conditions: role in embryo development, physiology and adaptation to the post-hatch environment. Front. Physiol..

[bib0043] Uni Z., Ferket P.R. (2004). Methods for early nutrition and their potential. Worlds Poult. Sci. J..

[bib0044] van der Wagt I., de Jong I.C., Mitchell M.A., Molenaar R., van den Brand H.A. (2020). Review on yolk sac utilization in poultry. Poult. Sci..

[bib0045] Willemsen H., Kamers B., Dahlke F., Han H., Song Z., Ansari Pirsaraei Z., Tona K., Decuypere E., Everaert N. (2010). High- and low-temperature manipulation during late incubation: effects on embryonic development, the hatching process, and metabolism in broilers. Poult. Sci..

[bib0046] Willemsen H., Li Y., Willems E., Franssens L., Wang Y., Decuypere E., Everaert N. (2011). Intermittent thermal manipulations of broiler embryos during late incubation and their immediate effect on the embryonic development and hatching process. Poult. Sci..

[bib0047] Yadgary L., Uni Z. (2012). Yolk sac carbohydrate levels and gene expression of key gluconeogenic and glycogenic enzymes during chick embryonic development. Poult. Sci..

[bib0048] Yair R., Shahar R., Uni Z. (2013). Prenatal nutritional manipulation by *in ovo* enrichment influences bone structure, composition, and mechanical properties. J. Anim. Sci..

[bib0049] Yalcin S., Özkan S., Shah T. (2022). Incubation temperature and lighting: effect on embryonic development, post-hatch growth, and adaptive response. Front. Physiol..

[bib0050] Zhai W., Gerard P.D., Pulikanti R., Peebles E.D. (2011). Effects of *in ovo* injection of carbohydrates on embryonic metabolism, hatchability, and subsequent somatic characteristics of broiler hatchlings. Poult. Sci..

